# Dumping Syndrome in Children: A Narrative Review

**DOI:** 10.7759/cureus.41407

**Published:** 2023-07-05

**Authors:** Mohammad Al-Jafari, Sallam Alrosan, Ibraheem M Alkhawaldeh, Sadeen Zein Eddin, Mohammad Abu-Jeyyab, Saja N Zuaiter

**Affiliations:** 1 School of Medicine, Mutah University, Al-Karak, JOR; 2 Internal Medicine, Saint Luke’s Health System, Kansas, USA

**Keywords:** continuous glucose monitoring, bariatric surgery, glucagon-like peptide 1, children, dumping syndrome

## Abstract

Dumping syndrome (DS) is a typical side effect of stomach surgery, which includes cancer, non-cancer esophageal and gastric surgery, and bariatric surgery. It is marked by the fast evacuation of undigested food from the stomach into the small intestine, which causes a variety of symptoms. Early dumping symptoms include gastrointestinal symptoms such as stomach discomfort, diarrhea, and nausea, as well as vasomotor symptoms such as drowsiness and face flushing, and occur within the first hour following a meal. Late dumping symptoms appear one to three hours after a meal and are related to reactive hypoglycemia, resulting in hypoglycemia, sweating, palpitations, and confusion.

Early dumping pathophysiology involves abnormalities in stomach structure and function, which result in rapid transit of stomach contents to the duodenum, insufficient digestion, and fluid transfers from the vascular compartment to the intestine. Late dumping occurs as a result of hyperinsulinemia caused by the fast passage of undigested foods to the gut. Symptom-based questionnaires and diagnostic testing such as plasma glucose measurement and stomach emptying studies can be used to confirm a diagnosis of DS.

The primary approach to managing DS is dietary modifications, including eating smaller, more frequent meals and avoiding high glycemic index carbohydrates. Dietary supplements and medications may be used to slow down gastric emptying or control blood glucose levels. Pharmacological options include alpha-glycosidase inhibitors, somatostatin analogs, glucagon-like peptide-1 analogs, and sodium-glucose cotransporter inhibitors. In severe cases, refractory to conservative measures, surgical interventions may be considered.

DS can arise in children following gastric surgery for obesity or corrective surgery for congenital abnormalities. It is frequently misdiagnosed and can have serious implications, such as hypoglycemia-related cognition deficits. Screening and early identification using glucose tolerance testing and continuous glucose monitoring (CGM) are critical in at-risk youngsters. Children's treatment techniques are similar to those used in adults, with dietary changes and medication therapies serving as the cornerstone of care.

Overall, DS is a complex condition that requires a multidisciplinary approach to diagnosis and management. Further research is needed to improve understanding of its pathophysiology and optimize treatment strategies, particularly in children.

This review aims to provide a well-rounded informative summary of the most recent literature on the under-recognized clinical and scientific aspects of DS among the children age group. It incorporates the quality of life, pathophysiology, diagnosis, prevalence, and treatment.

## Introduction and background

Dumping syndrome (DS) is a common side effect of cancer, non-cancer esophageal and gastric surgery, and bariatric surgery also known as metabolic surgery. These operations alter stomach structure and innervation, allowing a large volume of undigested food to enter the small intestine too quickly [1].

DS is a collection of symptoms that can be classified as early or late DS symptoms and can occur concurrently or independently [2]. Symptoms are grouped based on when they arise with the time from a meal. Early dumping symptoms include both gastrointestinal and vasomotor symptoms and occur during the first hour postprandially. Late dumping, on the other hand, occurs one to three hours after a meal and involves symptoms of reactive hypoglycemia [2,3].

Early symptoms comprise both gastrointestinal and vasomotor symptoms. Gastrointestinal symptoms include abdominal pain, diarrhea, borborygmi, nausea, and bloating. Vasomotor symptoms include fatigue, a desire to lie down after meals, facial flushing, palpitations, perspiration tachycardia, hypotension, and syncope. Late dumping symptoms include hypoglycemia, perspiration, palpitations, hunger, fatigue, confusion, aggression, tremor, and syncope [4].

The pathophysiology of early dumping includes gastric procedures such as sleeve gastrectomy, whole or partial gastrectomy, Nissen fundoplication, and esophageal surgeries, which are frequently associated with post-operative dumping symptoms. These procedures decrease the stomach's ability to retain food and eliminate the pylorus barrier function. This causes fast transit of stomach material to the duodenum, resulting in inadequate nutritional digestion. Because these nutrients are hyperosmolar, fluids migrate from the vascular compartment to the intestinal lumen. This can result in hypotension, dizziness, and syncope [5]. Furthermore, fluid redistribution promotes duodenal distension, which results in bowel contraction, diarrhea, and abdominal bloating. [6]. On the other hand, late dumping occurs due to a hyperinsulinemic state or reactive hypoglycemia [7]. Rapid transit of undigested nutrients to the intestine induces the release of a high amount of insulin into circulation in response to high glucose concentrations in undigested carbohydrates.

In individuals who are at risk of getting DS, the practitioner should be cautious about diagnosing it. Those with a history of surgery or who show classic DS symptoms are at risk of being diagnosed with this condition [8]. As a result, these individuals should investigate the possibility of DS. To confirm the diagnosis of DS among symptomatic individuals, three symptoms-based questionnaires are used: Sigstad score, Arts, and the dumping symptom rating scale [4,9]. Although the diagnostic value of plasma glucose measurement is modest, it can be utilized as a diagnostic test. However, if this test is performed on individuals who complain of late dumping symptoms, it may be more useful [10]. In addition, because it is a non-invasive treatment, the stomach emptying study provides an extra safe diagnostic tool [11]. Provocation tests may offer higher diagnostic sensitivity [8]. The oral glucose tolerance test (OGTT) and the mixed meal tolerance test are the two choices [9,12].

In general, the first and most important step toward minimizing the symptoms of DS is through setting the appropriate dietary program. Patients should be advised to eat less at each meal and divide the number of meals to six per day [8]. Another key nutritional measure that should be taken into account to ward off the hypoglycemic symptoms or the progression of DS is the restriction of the carbohydrate load and the choice of low glycemic index carbohydrates. Monomeric carbohydrate consumption must be replaced by high fiber and protein-rich foods as a substitute calorie source to compensate for the reduction of carbohydrate intake [9]. Lastly, as a last resort in dietary measures, lying down for half an hour after a repast would perpetuate gastric emptying and alleviate hypovolemic symptoms [4]. Dietary supplements including gum guar, pectin, and glucomannan can be added to the meals of an individual with DS [8]. These pills intend to increase the food viscosity, hence, slowing down the passage of food from the threshold of the stomach to the small intestines [13]. Gas formation and bloating are common intolerable side effects [8].

Moreover, pharmacological therapy can be considered a second-line intervention in patients not responding to dietary modifications. Medications including acarbose and somatostatin analogs may be introduced. Acarbose is an alpha-glycosidase inhibitor that functions to slow down the absorption of carbohydrates [14]. As its name denotes, it inhibits the α-glycosidase-mediated production of monosaccharides from carbohydrates in the epithelial brush border cells of the small intestine, thus, blunting postprandial hyperglycemia and subsequent hypoglycemia. The main adverse effect this drug holds is flatulence and bloating due to carbohydrate malabsorption.

Another drug that can be used is octreotide. It works by slowing gastric emptying and inhibiting the release of insulin and other gut hormones [13]. Octreotide comes in short and long-acting formulas. Short-acting formulations are administered subcutaneously three times daily while long-acting agents are given intramuscularly once every two to four weeks [4]. The main adverse events related to the use of somatostatin analogs are pain at the site of injection, gallstone formation, and nausea. Another common side effect is the occurrence of a mild form of steatorrhea [4,9]. A similar but more effective approach for improving control of postprandial hyperinsulinemic hypoglycemia after a gastric bypass is pasireotide [15]. It is a multi-receptor ligand second-generation somatostatin analog that has a 39-fold higher binding affinity for somatostatin receptor subtypes SST 1, 2, 3, and 5 [15]. The long-acting agent liraglutide and recombinant human glucagon-like peptide-1 (GLP-1) polypeptide beinaglutide induce inhibitory effects on gastric emptying, reducing insulin secretion within a short period of time after meals [16,17].

Recent trials have also suggested the role of intestinal sodium-glucose cotransporter-1 (SGLT-1) and sodium-glucose cotransporter-2 (SGLT-2) like canagliflozin in the pathophysiology of reactive hypoglycemia [18]. Both showed satisfactory performance in repressing symptoms of DS. SGLT-1 represents the primary pathway involved in intestinal glucose and galactose absorption. However, the main accepted mechanism for lowering blood glucose is by suppressing the renal SGLT-2 and, thus, increasing the urinary glucose excretion. It holds back glucose reabsorption in the proximal renal tubule, releases excessive glucose into the urine, and corrects the plasma glucose levels alleviating hypoglycemic symptoms [19,20]. On top of that, they work on reducing episodes of hypoglycemia by activating SGLT-1.

Other pharmacological interventions for treating symptoms of late DS include diazoxide. Its hyperglycemic activity is the result of an interplay with ATP-sensitive potassium channels found on the membrane of the pancreatic β cells [14]. This would allow sustained potassium efflux, thereby blocking the stimulation of the insulin release pathway. This drug has always been known to be an effective modality for treating pediatric age groups diagnosed with congenital hyperinsulinemic hypoglycemia [21]. Lastly, surgical options should be reserved for patients refractory to conservative and minimally invasive procedures. For most people, the type of surgery depends on the type of gastric surgery performed previously. However, surgery to correct DS often holds unsuccessful results.

This review claims to give a well-rounded comprehensive overview of the most recent research on the under-recognized clinical and scientific aspects of DS in children. It covers pathogenesis, diagnosis, prevalence, and therapy.

## Review

Prevalence

The prevalence of DS in children varies depending on the population studied and the surgical procedures performed [1]. Overall, DS is reported to occur in children who have undergone gastric surgeries, such as bariatric surgery or corrective surgeries for congenital anomalies. It is more commonly observed in children who have undergone surgeries for the treatment of obesity, such as sleeve gastrectomy or gastric bypass.

Furthermore, the prevalence of DS tends to increase following surgeries related to congenital anomalies, such as esophageal atresia repair or fundoplication.

DS has been long linked to a complication of fundoplasty with its variants (Nissen, Toupet). In a recent study, DS after Toupet fundoplication occurred in 17/190 patients (8.9%). Contrarily, reports state that up to 30% of Nissen operation patients experience DS. They believed that the chance of getting DS after Nissen’s fundoplication was either the same or greater than the risk following a Toupet fundoplication [22,23]. Despite being well-documented in adults, it frequently remains undiagnosed in children and has the potential to be lethal because many practitioners are unaware of this risk, and parents are rarely made aware of this potential problem. Additionally, unlike adults, children usually don't show as much evidence of early dumping, which results in symptoms including abdominal pain, bloating, nausea, vomiting, flushing, tachycardia, and hypotension which are frequently nonspecific but crucial to treat.

Additionally, hypoglycemia brought on by late DS can result in brain impairment, which can lead to major consequences like seizures, developmental delays, and other difficulties that can be avoided with early detection and treatment. Furthermore, the clinical significance of a short period of hyperglycemia remains unclear, particularly in the postsurgical period.

Further supporting the aforementioned points is the wide variation in incidence and clinical manifestations of DS among children reported in studies. For example, one institution noted that after implementing an asymptomatic hypoglycemia screening program, nearly 31 (24%) of 129 children who underwent fundoplication experienced postprandial hypoglycemia (PPH) within a week. Only two (1.3%) of 156 unscreened youngsters had PPH, in comparison [24]. The application of a screening program requires a great deal of labor and cost. To solve this problem, a growing body of research studied the risk factors of developing DS following fundoplication in children; thus, clinicians can only conduct postoperative screening among high-risk children. One study analyzed and identified risk factors, and it included fundoplication surgery within one year of age, the presence of severe scoliosis, microgastria, and major cardiac abnormality [25].

In short, the true incidence of DS in children remains controversial; some studies report it as an infrequent complication, while others estimate the frequency to be tremendous. This variability may be explained by differences in the definitions of DS and screening practices used in the diagnosis. While the exact incidence remains unknown, PPH remains a largely unrecognized complication following Nissen fundoplication [22]. It remains an underdiagnosed dilemma and strict follow-up is mandatory. For this, children at risk should go through an immediate screening during their postoperative period. Early detection and intervention of severe hypoglycemia will prevent poor neurocognitive outcomes and result in a better prognosis. Thus, a glucose tolerance test is indicated by even a single clinical complaint, usually feeding difficulties.

Although DS can arise following these surgeries, which are necessary to treat gastric esophageal reflux disease after surgical correction of esophageal atresia, it has been demonstrated that it can occur even without anti-reflux surgery in recent years [26,27]. Other very rare causes of DS in children resulting from abnormal GI anatomy include congenital microgastria, partial or total gastrectomy, accidental intraduodenal or jejunal administration of bolus feeding, gastrostomy or jejunostomy post pyloric bolus feeding in infants, inadequate meals with high osmolarity, and rare cases of generalized autonomic dysfunction [27-29]. The majority of these cases reveal that the gastric sphincter's tip has moved deeper into the duodenum. It is also infrequently referred to in children without a known underlying cause as idiopathic, unexplained, or isolated postprandial hyperinsulinemic hypoglycemia [30].

Pathophysiology

According to a study, children with PPH following Nissen fundoplication have greater plasma concentrations of the hormones GLP-1 and insulin and lower plasma glucose nadirs in response to an OGTT than controls. The authors hypothesized that elevated GLP-1 levels exacerbated the insulin spike and caused the consequent hypoglycemia. The study participants' normal fasting tolerance and proper suppression of insulin secretion in response to hypoglycemia negate the possibility of an underlying insulin secretion problem [31]. The quick increase in glucose and insulin levels could be explained by changes in the small intestine's ability to absorb nutrients as a result of the fundoplication, which reduces the stomach's capacity and accidentally damages the vagus nerve, which shortens transit time.

To more fully understand how endogenous GLP-1 contributes to PPH brought on by fundoplication, the insulin response to a standardized mixed meal in affected children was measured with concomitant administration of exendin-(9-39) or vehicle through a continuous intravenous infusion. This study discovered that the administration of the GLP-1 antagonist reduced the post-meal insulin spike, indicating that GLP-1 is crucial for the excessive insulin response that results in PPH in children who have had a fundoplication. In this investigation, exendin-(9-39) treatment resulted in a greater glucagon concentration than the vehicle condition [32].

Diagnosis

Continuous glucose monitoring (CGM) was introduced to be in recent years to be used as the first line of evaluation for children who may have hypoglycemia after GI surgeries because of the inconsistent outcomes in imaging investigations (gastric imaging studies, including gastric emptying studies, upper GI studies, and nuclear medicine studies) which showed to have a low diagnostic yield and the quick development of hypoglycemia unawareness in children. Intermittent glucometer checks like finger prick tests may miss abnormal blood sugar levels, and PPH in kids with a history of stomach surgery is probably underdiagnosed. Even if a kid has no or few clinical symptoms, clinicians should retain a high index of suspicion for DS when screening children who have had stomach surgery in the past. With CGM, DS can be identified early, extending the window of opportunity for diagnosis, which was previously thought to be the first week following the achievement of full feeding. For late dumping, the mean time after surgery was 27.6 months, and the pattern of glucose dysregulation is clearly visible. It can also be successfully used to evaluate how well various treatments and diet plans function. With CGM, the pattern of glucose dysregulation is very obvious, and DS can be identified early, extending the window of opportunity for diagnosis, which was previously thought to be the first week following the achievement of full feeding. For late dumping, the mean time after surgery was 27.6 months. It can also be successfully used to evaluate how well various treatments and diet plans function [24,33-36].

CGM helps direct treatment measures like feeding adjustments, cornstarch, and acarbose [33]. The use of dietary modification with more frequent or continuous feeds and dietary supplements with fiber, cornstarch, or gelling agents are the first-line treatments for PPH brought on by late DS. Fluid consumption and quickly digestible carbs during meals should be limited [4]. Acarbose, an alpha-glucosidase inhibitor that slows carbohydrate breakdown, is used as a second-line treatment. Diazoxide is also an option. Patients who do not improve after dietary changes and acarbose are given somatostatin analogs. The food and medication schedules were able to be modified safely thanks to CGM. Families were also able to recognize and respond to hypoglycemia episodes with CGM that could have gone undetected with conventional POC glucometer use. In the event of hypoglycemia, families are advised to administer a little amount of rapid-acting glucose [21,32,33].

Finally, a recent novel study investigated the eating habits of children born after maternal bariatric surgery, finding that maternal pre-pregnancy bariatric surgery does not alter unhealthy eating behaviors and the risk of development of childhood obesity in their children.

Their findings indicated the possibility of sugar-avoidance behavior in bariatric surgery children, fitting dietary maternal habits in a DS prevention strategy.

More research is needed to investigate the dietary habits of women after bariatric surgery, as well as the relationship between their offspring's dietary habits and the risk of developing DS [37].

Treatment

DS is a well-recognized complication after gastric surgery in adults. In recent years, studies recognized that DS may follow Nissen fundoplication in childhood. A number of factors such as carbohydrates, viscosity, and dietary fibers determine gastric emptying. Successful treatment of DS may be difficult to achieve. Yet, the appropriate management of DS should be aimed at resolving clinical symptoms and boosting nutrition. Similar to adults, a dietary regimen has proven itself to be a very effective form of treatment of symptoms resulting from DS in children. The dietary changes include a decrease in the amount of liquids and simple carbohydrates, an increase in fat content, and the administration of smaller and more frequent feedings [38]. However, these measures can be quite challenging in children who are unable to eat by mouth. Instead, they can be fed exclusively by gastrostomy with specialized liquid diets directly installed into the antrum. These enteral diets pose a challenging task. They are expensive and may not be readily available, and whenever carbohydrate concentrations are decreased to alleviate the signs and symptoms, growth is usually hampered due to insufficient caloric intake. As a result, an alternative to control DS is to decrease the amount of food and glucose delivered to the stomach at each meal and in return increase the intake of solid food. One study showed that particular dietary modifications included capping the daily carbohydrate intake to approximately 2 g/kg. Several children were also given a carbohydrate-free formula supplemented with 2 g/kg/day fructose; when extra calories were required, medium-chain triglycerides were added [29]. Another study by Samuk et al. proved that with three months of dietary compliance, there was clinical improvement in 13 of 14 patients, while after six months, there was all 14 patients had clinical improvement [22].

Uncooked corn starch has been advocated by several authors as an effective addition to ameliorating the worsening of symptoms [38,39]. Uncooked cornstarch is a complex carbohydrate composed of highly branched glucose chains. It is gradually hydrolyzed in the small intestine, and thus, when given in the form of a bolus, it would provide a continuous source of glucose that is slowly absorbed into the bloodstream, hence delaying gastric emptying [38]. A study published in 1998 revealed that the modification of feedings and the addition of uncooked starch improved the quality of life of patients suffering from DS. It resulted in the normalization of symptoms and glucose values especially those fed exclusively by gastrostomy [38]. As long as uncooked starch was the sole dietary carbohydrate, the reversal of dumping symptoms correlated with the elimination of postprandial hyperglycemia and hyperinsulinemia. The effect lasted as long as uncooked starch was given.

Moreover, several authors have reported the positive effects of decreasing carbohydrates and increasing the fat content of feeds through the utilization of corn oil, lipid suspensions, and medium-chain triglyceride [39,40]. According to Khoshoo et al., dietary manipulation was built in accordance with the altered physiology following gastric surgery. Consequently, the correction of the blood glucose abnormalities of the two children who underwent Nissen's fundoplication, resolution of symptoms, and weight gain were effectively achieved by the addition of fats and uncooked corn starch to their feeds [40]. Fats were used to delay gastric emptying and concurrently uncooked corn starch was used to deliver small amounts of glucose at a steady rate over a relatively longer time period. Although the current regimen of uncooked starch proved an effective means of controlling the symptoms, enhancing calorie intake, weight gain, and oral feedings, enabling a more normal lifestyle, maximizing developmental potential, and providing a better quality of life, there are no precise guidelines for its utilization, and no specific information about its use in children fed exclusively by gastrostomy [22,41].

A second-line therapy is acarbose, an alpha-glucosidase inhibitor that functions to blunt the intestinal absorption of carbohydrates resulting in the reduction of postprandial hyperglycemia in both type 1 and type 2 diabetes mellitus. Meanwhile, somatostatin analogs are reserved for patients who do not respond to dietary manipulations and acarbose [33]. Overall, there are no reports of pediatric cases managed with pectin, insulin, tolbutamide, or somatostatin nor have surgical procedures been used to delay gastric emptying [39]. One study portrayed that the blockade of the GLP-1 receptor is effective in reducing the insulin surge seen in children with PPH. Accordingly, the possibility of new targeted therapies to decrease the effects of GLP-1 may upgrade the management of these children in the upcoming years [32].

## Conclusions

In conclusion, DS is a complex illness that can develop in children who have undergone stomach surgery, such as bariatric surgery or corrective surgery for congenital problems. The frequency of DS varies depending on the population studied and the type of surgery performed. It is more commonly found in children who have had obesity treatment or surgery to treat congenital defects. Because it produces symptoms such as stomach pain, bloating, nausea, vomiting, flushing, tachycardia, and hypotension, DS can have major effects on children's health. In extreme cases, it can also cause hypoglycemia, which can lead to brain malfunction and developmental delays. Unfortunately, DS is commonly misdiagnosed in children, and many healthcare practitioners are unaware of the risks. Because traditional screening tests have a low diagnostic yield, diagnosing DS may be challenging. CGM, on the other hand, has emerged as an important tool for the early detection and monitoring of DS in children. CGM detects abnormal glucose patterns and alerts management to avert negative consequences. The primary therapy for DS in children is dietary adjustments. Reduced liquid and simple carbohydrate consumption, higher fat content, and smaller and more frequent feedings can all help reduce symptoms. Uncooked maize starch has been discovered to be an effective dietary supplement, providing a slow and continuous release of glucose while delaying stomach emptying. In some cases, acarbose or somatostatin analogs may be used as second-line therapy. More research is needed to better understand the prevalence of DS in children, risk factors, and effective management techniques. Furthermore, long-term impacts and prospective preventive interventions, such as maternal dietary habits, require investigation. Overall, DS diagnosis and treatment in children is crucial for their long-term development and well-being. To improve outcomes and offer a better quality of life for affected children, prompt diagnosis, appropriate management, and close monitoring are essential.

## Appendices

Abbreviations

DS: dumping syndrome

OGTT: oral glucose tolerance test

SGLT-1: sodium-glucose cotransporter-1

SGLT-2: sodium-glucose cotransporter-2

PPH: postprandial hypoglycemia

GLP-1: glucagon-like peptide-1

CGM: continuous glucose monitoring
